# 食管破裂致脓毒症合并弥散性血管内凝血及肝功能障碍1例

**DOI:** 10.3760/cma.j.cn121090-20241209-00548

**Published:** 2024-12

**Authors:** 健 张, 晓春 马

**Affiliations:** 中国医科大学附属第一医院重症医学科，沈阳 110001 Critical Care Medicine, the First Affiliated Hospital of China Medical University, Shenyang 110001, China

患者，男，45岁，2021年3月15日因“剧烈呕吐后胸痛伴呼吸困难1 d”入院。患者2021年3月14日饮酒、饱食后剧烈呕吐，呕吐后突发左侧胸部剧烈疼痛，随呼吸加重，伴呼吸困难。于中国医科大学附属第一医院急诊行增强CT检查示：食管穿孔，左侧液气胸，右侧胸腔积液。2021年3月16日于急诊行开胸探查、食管破裂修补术、胸腔闭式引流术，手术时长约3 h。术中静脉输注晶体液1 500 ml，胶体液1 000 ml，碳酸氢钠注射液250 ml。尿量450 ml，失血200 ml，术中血流动力学不稳定，存在感染性休克。转入ICU初期SOFA评分11分，APACHE Ⅱ评分23分。生命体征：体温37.9 °C，血氧饱和度99％，心率122次/分，血压102/56 mmHg（去甲肾上腺素1.1 µg·kg^−1^·min^−1^）。机械通气：模式PC，吸入氧浓度60％，吸气压力16 cmH_2_O，呼气末正压8 cmH_2_O，呼吸频率16次/min。氧合指数140 mmHg。血常规：WBC 2.21×10^9^/L，中性粒细胞百分比65.5％，HGB 148 g/L，PLT 86×10^9^/L。凝血功能：PT 28.1 s，凝血酶原活动度（PTA）28％，APTT 60.2 s，D-二聚体2.15 µg/ml（FEU），纤维蛋白（原）降解产物（FDPs）6.83 µg/ml，纤维蛋白原4.57 g/L，At-Ⅲ活性45％；感染指标降钙素原（PCT）>100 ng/ml，C反应蛋白（CRP）161.5 mg/L。心肺超声示心脏整体收缩力增强，下腔静脉1.3 cm，变异度明显。双侧上下蓝点为A线，右侧膈肌点碎片征，PLAS点组织样变及胸腔积液，左侧PLAS点及膈肌点见B7线及少量胸腔积液。予抗感染、抗休克治疗，液体复苏2 h后效果不佳，血流动力学恶化，超声提示左心增大，左室心肌弥漫性收缩能力下降，射血分数（EF）30％。应用左西孟旦强心，联用垂体后叶素维持血压，盐酸艾司洛尔注射液控制心率，加用乌司他丁和血必净注射液改善微循环，行高流量血液滤过减轻应激，PICCO监测血流动力学。经上述治疗后患者血流动力学好转，胃肠减压可见淡血性胃液，尿管可见肉眼血尿，皮肤黏膜未见明显出血。复查血常规WBC 3.32×10^9^/L，中性粒细胞百分比67.8％，HGB 117 g/L，PLT 86×10^9^/L；肝功能：ALT 1 068 U/L，ALP 35 U/L，GGT 23 U/L，总蛋白38.9 g/L，白蛋白23.3 g/L，总胆红素50 µmol/L，直接胆红素15.9 µmol/L；凝血功能：PT 40.5 s，PTA 18％，APTT 180 s，D-二聚体2.47 µg/mL（FEU），FDPs 7.96 µg/ml，纤维蛋白原3.06 g/L，At-Ⅲ活性30％。上述结果提示肝功能异常，凝血因子不足，予维生素K肌肉注射补充凝血因子底物，患者存在出血倾向，预约血浆400 ml，加用还原型谷胱甘肽、多烯磷脂酰胆碱保肝治疗。满足国际血栓止血学会和日本急救医学会的弥散性血管内凝血（DIC）诊断标准，诊断为脓毒症合并DIC。予普通肝素20 mg持续静脉泵入，血液滤过的抗凝方式由枸橼酸钙改为普通肝素。经上述治疗后患者休克纠正，心肌抑制解除，肝肾功能逐步稳定，2021年3月19日撤离机械通气，拔除气管插管。2021年3月22日转出ICU。

讨论：自发性食管破裂是一种严重的胸外科急症，胸内负压不仅可引起空气和胃液被吸入纵隔和胸腔，还可造成化学性胸膜炎和纵隔炎，引发严重的脓毒症，具有极高的死亡率。本例患者在食管破裂后迅速发生了严重的感染性休克及多脏器功能衰竭，且治疗过程中出现了明显的出血倾向，仅依靠脓毒症伴DIC并不能完全解释其出血表现。当前普遍认为脓毒症相关凝血病是从免疫血栓转变为血栓过度形成的过程，但ICU患者常处于血栓过度形成阶段。重症患者的凝血功能异常可能由多种病因交替作用，本例患者的凝血功能改变与脓毒症伴DIC及肝功能损伤引起的凝血因子合成障碍密切相关。凝血酶在DIC的发病机制中发挥着关键作用，肝素能够通过抑制凝血酶的活性有效调节凝血过程。因此，使用肝素治疗脓毒症合并DIC具有重要的临床意义。有临床研究发现，肝素治疗脓毒症合并DIC患者28 d病死率更低。对于有明显出血倾向或肝脏合成凝血因子能力下降的患者，输注血制品可能是恰当的。本病例在治疗过程中采用肝素抗凝，并通过对症补充凝血因子有效改善了患者的病情。提示在重症患者的凝血治疗中，需在纠正病因的基础上，结合患者的病理生理特点，选择合适的干预方案。

